# A Phase I clinical and pharmacological study of *cis*-diamminedichloro(2-methylpyridine) platinum II (AMD473)

**DOI:** 10.1038/sj.bjc.6600854

**Published:** 2003-04-01

**Authors:** P Beale, I Judson, A O'Donnell, J Trigo, C Rees, F Raynaud, A Turner, L Simmons, L Etterley

**Affiliations:** 1 Sydney Cancer Centre, Royal Prince Alfred Hospital, Missenden Road, Camperdown, Sydney, New South Wales, Australia; 2 The Cancer Research UK Centre for Cancer Therapeutics, Institute of Cancer Research, 15 Cotswold Rd Belmont Sutton, Surrey SM2 5NG, UK; 3 Hospital Universitari de la Vall d'Hebron, Passeig de la Vall d'Hebron, Barcelona, Spain; 4 Cancer Research UK, PO Box 123, Lincoln's Inn Fields, London WC2A 3PX, UK

**Keywords:** phase I, AMD473, platinum, clinical trial

## Abstract

AMD473 is a novel sterically hindered platinum cytotoxic with demonstrated ability to overcome acquired resistance to cisplatin *in vitro* and in human tumour xenografts. A single-agent dose escalating Phase I study was performed. AMD473 was initially administered intravenously as a 1 h infusion every 21 days to patients with advanced solid tumours. In total, 42 patients received a total of 147 cycles (median 3, range 1–8) of treatment at doses of 12, 24, 48, 96, 110, 120, 130, and 150 mg m^−2^. Dosing intervals of 21 and 28 days were explored at the recommended dose. Neutropenia and thrombocytopenia proved dose limiting. Other toxicities included moderate nausea, vomiting, anorexia, and a transient metallic taste. There was no significant alopecia. The maximum tolerated dose was 150 mg m^−2^. Plasma pharmacokinetics were linear. Two patients with heavily pretreated ovarian cancer showed partial response. Five patients (mesothelioma, ovary, nonsmall cell lung, and melanoma) showed prolonged stable disease. AMD473 demonstrates encouraging activity in patients, including those with prior platinum exposure. Toxicity is predictable with linear pharmacokinetics, as was predicted by preclinical studies. A dose of 120 mg m^−2^ every 21 days is recommended for Phase II evaluation although there is evidence that chemo-naive patients and those of good performance status may tolerate a higher dose.

While cisplatin is used widely in the clinic to treat many tumour types, clinical utility is limited by two major factors: (i) drug resistance, either intrinsic or acquired, and (ii) the principal dose-limiting side effects of nephrotoxicity and neurotoxicity. Unfortunately, although the initial tumour response to cisplatin in many tumours may be high, most patients will nevertheless relapse and die of their disease.

AMD473 was primarily designed to overcome one of the known mechanisms of platinum resistance: detoxification by intracellular thiols, through the introduction of a bulky methylpyridine ring to provide steric hindrance to direct interaction with platinum (Figure 1

**Figure 1:**
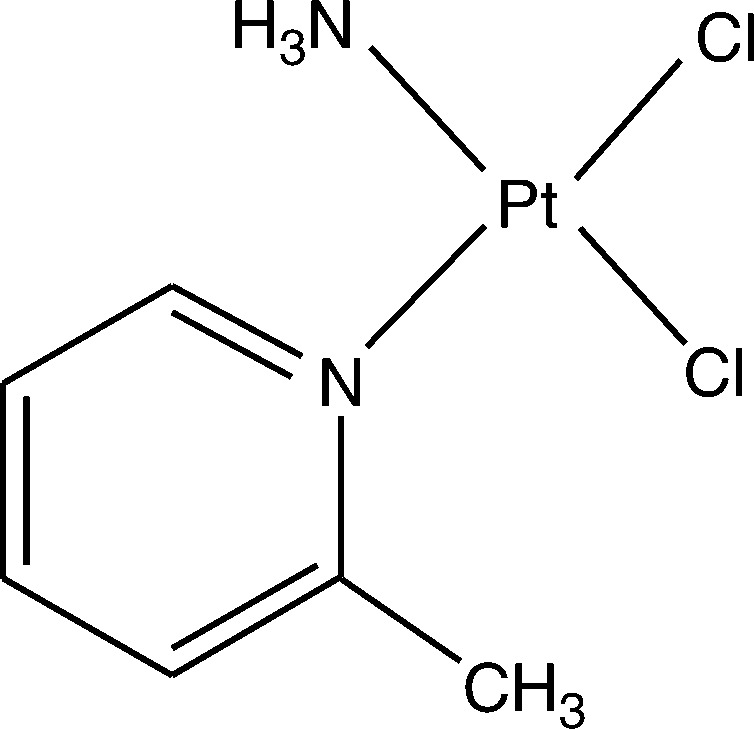
AMD473 structure.

) (Raynaud *et al*, 1997). Preclinical evaluation of AMD473 confirmed that this drug was also able to overcome platinum complex resistance in cell lines with high levels of glutathione (GSH) (Holford *et al*, 1998a,1998b). In studies with human ovarian cell lines, it has been shown that increasing levels of reduced GSH are associated with increasing platinum resistance (Mistry *et al*, 1991). In addition, lower levels of glutathione S-transferase (GST) activity have been shown to be associated with enhanced clinical response to platinum-based chemotherapy in head and neck cancer (Nishimura *et al*, 1996). Furthermore, AMD473 was able to overcome resistance because of decreased cellular uptake of drug in some cell lines or enhanced DNA repair/increased tolerance of platinum adducts in others (Holford *et al*, 1998a,1998b). AMD473 forms interstrand cross-links, but does so at a rate intermediate to that of cisplatin and carboplatin, because of its reduced reactivity relative to cisplatin (Holford *et al*, 1998a,1998b). Using a *Taq* polymerase stop assay to identify the site of DNA adducts, a novel pattern of DNA binding was identified in pBR322 DNA after incubation with 10 and 100 μM AMD473 for 2 h (Holford *et al*, 1998a). This finding may also in part account for the observed capability of AMD473 to circumvent adduct tolerance and DNA-repair mechanisms associated with resistance to cisplatin.

Preclinical pharmacology studies in mice have demonstrated that the maximum tolerated dose (MTD) is 45 mg kg^−1^ given as a single intraperitoneal administration, with the dose-limiting toxicity (DLT) being myelosuppression. Owing to the limited aqueous solubility of AMD473, preclinical toxicology studies were conducted by intraperitoneal injection following a pharmacokinetic demonstration of equivalent bioavailability of intraperitoneal and intravenous administration. No renal toxicity was observed (Pre-clinical toxicology report – CR UK, Drug data file, Q11997).

Antitumour activity was noted in several tumour models including human ovarian carcinoma xenografts with acquired resistance to cisplatin (CH1cisR) and carboplatin (HX110P). In head-to-head experiments, AMD473 showed an improved antitumour effect compared with cisplatin and satraplatin (JM216, bis-acetato-ammine-dichloro-cyclohexylamine platinum IV) against the CH1cisR xenograft (34 days growth delay *vs* 10.4 and 3.5 days, respectively). The antitumour activity was similar when AMD473 was given daily for 5 days every week for 4 weeks (60 mg kg^−1^ for 4 weeks) or by weekly administration (300 mg kg^−1^ every 7 days for 4 weeks) (Raynaud *et al*, 1997).

On the basis of the preclinical antitumour activity seen with AMD473, especially in models with acquired platinum resistance, the lack of nonhaematological toxicity, and the activity via the oral route, the drug was taken into clinical development. This Phase I trial commenced at the Royal Marsden Hospital under the auspices of the Cancer Research UK Phase I/II Committee. The initial schedule chosen was a short intravenous infusion given once every 3 weeks. A pharmacokinetically guided dose escalation scheme was used, for several reasons. Firstly, there is no evidence of metabolism of AMD473 *in vivo* and thus the free platinum AUC (area under the concentration *vs* time curve) should reflect the biologically important species in both man and mouse. Secondly, the previous experience with cisplatin and carboplatin has demonstrated the close relation between the AUC at MTD in the mouse and the AUC in humans at MTD (Van Hennik *et al*, 1987). It was hoped that this approach would reduce the number of dose escalations required to reach MTD.

## METHODS

### Study design

The trial was an open-label, dose-escalating, single-centre Phase I trial. The study protocol and patient information sheet were approved by the Royal Marsden Research Ethics Committee. The study was managed and conducted in accordance with the principles of the International Committee on Harmonisation Good Clinical Practice and with the Cancer Research UK's Standard Operating Procedures. The objectives of the trial were to determine (1) the MTD, (2) the DLTs, (3) the pharmacokinetics in humans, (4) evidence of antitumour activity, and (5) pharmacokinetic and pharmacodynamic relations when the drug was administered as a short infusion intravenously over 1–2 h, depending on the volume. The starting dose was 12 mg m^−2^ which was one-tenth of the MTD/lethal dose in 10% of mice (LD_10_) (Raynaud *et al*, 1997).

### Study population

All patients were required to have a pathologically confirmed diagnosis of a malignant solid tumour proven refractory to conventional treatment or for which no conventional treatment is standard. Additional eligibility criteria included a WHO performance status of 0–2, a life expectancy of greater than 3 months, and age ⩾18 years. Previous anticancer chemotherapy had to be discontinued for at least 4 weeks prior to entry into the study or 6 weeks in the case of pretreatment with mitomycin-C or nitrosureas. Patients were required to have recovered from all toxicity arising from prior therapy, with no evidence of serious concomitant medical problems or hearing loss, and should have completed any large field radiotherapy at least 4 weeks before study entry. All patients had to have acceptable bone marrow, liver and renal function as defined by WBC ⩾3.0 × 10^9^ l^−1^, neutrophil count ⩾1.5 × 10^9^ l^−1^, platelet count ⩾100 × 10^9^ l^−1^, bilirubin ⩽25 μmol l^−1^; ALT, ALP ⩽1.25 × ULN or ⩽5 × ULN in the presence of metastases and a creatinine clearance (as measured by ^51^Cr EDTA clearance or calculated according to the method of Cockcroft and Gault (1976)). All patients were asked to sign written informed consent prior to participation.

### End points

The treatment was to be given every 3 weeks and patients could continue to receive further cycles while there was no evidence of disease progression or significant toxicity. Dose escalation was to be performed according to the pharmacological guided dose escalation method based on the ultrafiltrable AUC. Using this method, dose levels would be doubled until the AUC of ultrafiltrable platinum was ⩾40% of the mouse MTD in two out of three patients, or there was grade II drug-related toxicity (not including nausea or vomiting and alopecia) seen in >1 patient at a particular dose level. Thereafter, dose escalations would be according to the modified Fibonacci method (60, 40, 30%) until MTD was reached (Schneiderman in Simon, 1993). At each dose, three patients would be treated unless a need for expansion was indicated, for example, drug-related grade II organ specific toxicity or grade III antiproliferative toxicity (myelosuppression, muco-sitis, or diarrhoea).

The initial patient at each dose level was observed for 3 weeks prior to further patients being enrolled. Escalation to the next dose level could only proceed when all three patients at the previous level had been observed for 3 weeks. Full pharmacokinetic data were reviewed prior to each dose escalation. The MTD was defined as the dose at which three or more of six patients suffered DLT. In turn, DLT was defined as the occurrence of: grade 4 neutropenia of ⩾5 days duration; other grade 3 antiproliferative toxicities such as thrombocytopenia, neutropenic sepsis, diarrhoea requiring hydration; or ⩾grade 2 organ-specific toxicity. The dose recommended for the Phase II study would then be chosen, either the dose prior to that defined as MTD or an intermediate dose. Provided myelosuppression proved dose-limiting the Phase II dose would be defined as that which could be delivered safely and repeatedly without significant incidence of grade 4 thrombocytopenia or grade 4 neutropenia of more than 5 days duration.

### Study drug

AMD473 was supplied as a sterile 0.4 mg ml^−1^ solution in 0.9% saline, as 200 mg (500 ml) bags or 10 mg (25 ml) ampoules, by the Cancer Research UK Formulation Unit, Department of Pharmaceutical Sciences, University of Strathclyde. The drug solution was further diluted in 0.9% saline for administration. At doses up to 200 mg, 500 ml was infused, and for doses greater than 200 mg, 1000 ml was infused with treatment given over an hour independent of volume. The final reconstituted formulation at concentrations of 0.4 mg ml^−1^ or lower is stable for at least 6 months when kept at room temperature and protected from light.

### Pharmacokinetic studies

Pharmacokinetic blood sampling was performed in all patients during the first course of treatment. Blood was collected into heparinised tubes for total and ultrafiltrable platinum analysis at time zero, 30 min, 1 and 2 h into the infusion; and then 5, 15, 30, 45, 60 min, 2, 4, 6, 9, 12, 24, 48, 72 h, and day 8 after infusion. The samples were centrifuged at 2000 **g** for 5 min at 4°C to prepare the plasma. Two aliquots were stored for total platinum analysis in plasma and plasma ultrafiltrates were subsequently prepared using two Amicon Centrifree Filters (30 000 MW cutoff) per sample. The filters were centrifuged at 2000 **g** for 20 min at 4°C and the ultrafiltrates stored in liquid nitrogen prior to analysis. Urine was collected for three 12-h time periods until 36 h after the infusion started. The volumes were recorded and aliquots were stored frozen for platinum analysis. Pharmacokinetic analysis was performed using WinNonlin (Pro model 202) using noncompartmental analysis.

### Toxicity and response assessment

Toxicity was assessed at the visits to the clinic using the National Cancer Institute of Canada Clinical Trials Group (NCIC-CTG) Common Toxicity Criteria V1 and supplemented through the use of daily diary cards. Patients were assessed for clinical response prior to each treatment and with radiological evaluation every two cycles. Responses were graded according to the WHO criteria for response (WHO, 1979). Audiometry and creatinine clearance (according to the method of Cockcroft and Gault) was reviewed prior to each cycle of treatment.

## RESULTS

### Patient accrual

In total, 42 patients were entered in the study between 10 November 1997 and 14 December 1999. The patients' demographic and baseline characteristics are listed in Table 1

**Table 1:** Patient characteristics

Patient no.	42
Gender (M/F)	16/26
Age (median range)	53 (33–71)
*PS (WHO)*	
0	3
1	22
2	17
*Tumour type*	
Ovary	10
Nonsmall cell lung	9
Mesothelioma	7
Head and neck	6
Colon	3
Other	7
*Prior treatment*	
Prior radiotherapy	19 (45%)
Prior chemotherapy	41 (98%)
Prior hormonal, biological or immunotherapy	15 (38%)
Previous platinum	37 (88%)

. The number of patients assigned to each dose level is shown in Table 2

**Table 2:** Dose escalation – listing of dose levels treated, cycle length, and incidence of DLT

**Dose (mg m^−2^)**	**Patient number**	**Duration of one cycle (days)**	**Number of patients experiencing DLT**
12	3	21	0
24	3	21	0
48	3	21	0
96	3	21	0
130	5	28	3
110	5	28	0
120	3	28	0
150	6	28	3
120	11	21	1

. A total of 147 cycles were administered and the median number of cycles was three (range 1–8). In all, 41 (98%) patients had received prior chemotherapy before entering the study. The median time (exclusive) between the last chemotherapy end date and the first cycle start date was 22 weeks (range of 4–86 weeks). Of these 41 patients who had received prior chemotherapy, the median number of regimens per patient was four. In all, 37 patients had received prior platinum chemotherapy: 14 patients started their first cycle of study drug treatment less than 6 months after stopping platinum chemotherapy, 12 patients within 6 months to 1 year, and 11 patients after more than 1 year.

### Toxicity

From a starting dose of 12 mg m^−2^, the dose of AMD473 was doubled until grade 2 drug-related haematological toxicity was observed in one out of three patients treated at 96 mg m^−2^. At this dose, the AUC for free platinum was >40% of the AUC of free platinum observed in mice at the MTD. Therefore, the treatment dose was escalated to 130 mg m^−2^, which correlated with a projected free platinum AUC equivalent to that at the mouse MTD. At this dose, DLT was observed in three patients; hence MTD was reached in this cohort. Treatment was planned to be delivered once every 3 weeks, but in the patient group recruited at 130 mg m^−2^ it could only be delivered every 4–5 weeks because of myelosuppression (predominantly thrombocytopenia). It was therefore decided to explore intermediate doses. At 110 mg m^−2^ (every 4 weeks), no DLT was observed in five patients leading to re-escalation to 120 mg m^−2^ (every 4 weeks), where again DLT was not seen in three patients.

It was noted that the patients who had received the study drug at dose level 130 mg m^−2^ were very heavily pretreated and some had borderline performance status. Given the lack of toxicity in patients at dose levels 110 and 120 mg m^−2^, a further six patients were enrolled at 150 mg m^−2^ (every 4 weeks) who had not been heavily pretreated (no more than two previous cycles of chemotherapy or one course of carboplatin) and who had a good performance status. At this dose level, three of six patients developed DLT, indicating a definite MTD. Further patients were subsequently recruited at the 120 mg m^−2^ dose level, using the original 3 weekly interval. Thus, 14 patients in total were treated at 120 mg m^−2^: 11 at a 3 weekly interval and three patients who received the drug at a 4 weekly interval.

In all, 15 patients had significant treatment delay or dose reductions because of drug-related toxicities, primarily myelosuppression, at dose levels of 96 mg m^−2^ or above. The grade 3/4 haematological toxicities, as seen in cycle one as well as overall, are listed in Table 3

**Table 3:** Dose escalation – listing of dose levels treated, cycle length, and incidence of DLT

**(a) Incidence during the first cycle**						
	**Dose level, total patients treated at this dose level**					
	**12, 24, 48 mg m^−2^**	**96 mg m^−2^**	**130 mg m^−2^**	**110 mg m^−2^**	**150 mg m^−2^**	**120 mg m^−2^**
**Toxicity**	***N*=9**	***N*=3**	***N*=5**	***N*=5**	***N*=6**	***N*=14**
Haematological						
Anaemia	0	0	1	0	0	0
Thrombocytopenia	0	0	2**	0	3***	0
Leucopenia	0		2	0	2	0
Neutropenia	0	0	2	0	3	0
Neutropenic sepsis	0	0	0	0	0	0
Lymphopenia	0	1	2	1	0	3
*Nonhaematological*						
Emesis	0	1	0	1	0	0
Malaise	0	0	0	0	0	1*
**(b) Incidence overall**						
**Dose**	**Total number of cycles**	**WCC, number of cycles affected (%)**	**Neutrophils, number of cycles affected (%)**	**Platelets, number of cycles affected (%)**	**Hb, number of cycles affected (%)**	
12	11	0 (16.7)	0 (16.7)	0 (16.7)	0 (16.7)	
24	10	0 (16.7)	0 (16.7)	0 (16.7)	0 (16.7)	
48	9	0 (16.7)	0 (16.7)	0 (16.7)	0 (16.7)	
96	12	1 (16.7)	1 (16.7)	1 (16.7)	1 (16.7)	
110	19	1 (16.7)	3 (15.8)	2 (10.5)	0 (16.7)	
120	48	2 (4.2)7	5 (10.4)	6 (12.5)	2 (4.2)7	
130	12	2 (16.7)	2 (16.7)	3 (25)7	1 (8.3)7	
150	26	4 (15.4)	6 (23.1)	12 (46.2)	3 (11.5)	

* Each represents a DLT event.

. Thrombocytopenia was the most commonly observed toxicity in 30 patients (71%) who experienced 103 episodes of thrombocytopenia, of which 24% were ⩾grade 3. At 110, 120, 130, and 150 mg m^−2^, thrombocytopenia grade 3 or 4 occurred in 10, 12, 25, and 46% of cycles. One patient, who had adenocarcinoma of the ovary, experienced fatal grade 4 rectal bleeding after receiving her second cycle of the study drug. After an initial cycle of treatment at 130 mg m^−2^, during which she required a platelet transfusion for grade 4 thrombocytopenia, she went on to receive a second cycle at the reduced dose of 96 mg m^−2^. Grade 4 rectal bleeding associated with grade 4 thrombocytopenia, started 12 days after administration of this second cycle and the patient died. There was evidence of progressive disease and no leukopenia; hence it was believed that thrombocytopenia had occurred either because of disseminated intravascular coagulation or simply because of consumption as a result of persistent bleeding.

Neutropenia was seen at all the higher dose levels with 16, 10, 17, and 23% of cycles complicated by grade 3 or 4 neutropenia at 110, 120, 130, and 150 mg m^−2^, respectively. In all, 34 patients (81%) experienced lymphocytopenia, and of these episodes 49% were ⩾grade 3. Anaemia was generally mild (grades 1 and 2) and tended to occur in later cycles, suggesting a cumulative effect.

None of the patients treated at the two lowest doses (i.e. 12 and 24 mg m^−2^) received prophylactic antiemetics, although two out of three patients at 24 mg m^−2^ required symptomatic treatment. One of three patients at 48 mg m^−2^, and two of three patients at 96 mg m^−2^, received prophylactic antiemetics. At 110 mg m^−2^ and above, prophylactic antiemetics were given routinely. Nausea (62% of patients), vomiting (36% of patients), and anorexia (31% of patients) were the most frequent gastrointestinal side effects. Nausea and vomiting were generally controlled by granisetron 1 mg with dexamethasone 8 mg prior to therapy, although some patients did require supplemental metoclopramide or dexamethasone for delayed symptoms.

No drug-related nephrotoxicity was observed, although two patients developed a rise in creatinine because of ureteric obstruction secondary to pelvic disease. Changes in the ECG were seen in two patients after one and three cycles, respectively, but were not clinically significant. One patient with nonsmall cell lung cancer developed grade 3 elevation of bilirubin after two cycles of treatment, which was possibly related to AMD473. Nine episodes of infection in six patients were considered to be drug-related, including four episodes of candidiasis, two episodes of cellulitis of the left neck, two chest infections, and one urinary tract infection. Only one of these occurred in the context of neutropenia. Malaise and fatigue were reported by 21 patients (50%) and 70% of these episodes were considered almost certainly, probably or possibly related to the treatment. Hypokalemia was reported in 18 patients, and considered to be drug-related in six (one at 120 mg m^−2^ and five at 150 mg m^−2^). All episodes were grade 1 or 2 and all resolved. No reproducible significant changes in audiometry were seen. In all, 14 episodes of grade 1/2 abnormal taste sensation occurred in seven patients with an onset time of between 7 and 21 days after dosing. At the two highest dose levels (120 and 150 mg m^−2^), the mean number of cycles received was 3.5, with three patients receiving four cycles and four patients receiving six cycles. There was no evidence of sensory or motor neuropathy.

### Pharmacokinetics

Platinum levels were measured in plasma, plasma ultrafiltrate, and urine by atomic absorption spectrophotometry using a previously validated method adapted for clinical use (Raynaud *et al*, 1997). The lower limit of quantification (LLQ) of the pharmacokinetic assay was 5 ng ml^−1^. The mean values for the pharmacokinetic parameters measured are presented in Table 4

**Table 4:** Dose escalation – listing of dose levels treated, cycle length, and incidence of DLT

**Dose (mg m^−2^)**	***C*_max_ mean (*μ*g l^−1^)**	**s.d.**	**AUC_last_ mean (h *μ*g l^−1^)**	**s.d.**	**AUC_inf_ mean (h *μ*g l^−1^)**	**s.d.**	***t*_1/2*γ*_ mean (h)**	**s.d.**	**Cl_obs_ mean (l h^−1^)**	**s.d.**	**V_ssobs_ mean (l)**	**s.d.**
12	353	48	896	522	985	655	12.3	18.6	13.9	7.0	78.6	88.5
24	676	90	1284	305	1308	322	3.2	3.1	17.7	3.6	37.5	6.5
48	1216	203	2598	148	3062	175	31.9	15.1	15.2	0.8	303.1	153.4
96	3197	326	7170	1214	7642	1359	50.9	14.5	12.1	3.4	286.3	43.6
110	3142	890	7919	2436	8717	2765	49.3	28.5	8.3	1.6	369.1	260
120	3250	647	11 139	5183	13 078	6360	85.7	38.9	12.4	3.9	560.3	340.3
130	3636	955	10 407	1441	13 318	3241	96.5	63.6	10.2	4.2	502.4	342.8
150	3740	1101	12 604	3713	13 960	4301	67.5	25.2	11.0	3.8	462.2	70.6

s.d.=standard deviation, AUC_last_=area under the dose concentration curve to last time point, AUC_inf_=area under the dose concentration curve extrapolated to infinity, *t*_1/2*γ*_=terminal half-life, Cl_obs_=observed clearance, V_ssobs_=volume of distribution at steady state.

and Table 5

**Table 5:** Dose escalation – listing of dose levels treated, cycle length, and incidence of DLT

**Dose**	***C*_max_ mean (*μ*g l^−1^)**	**s.d.**	**AUC_last_ mean (h *μ*g l^−1^)**	**s.d.**	***t*_1/2*γ*_ mean (h)**	**s.d.**	**Cl_obs_ mean (l h^−1^)**	**s.d.**	**V_ssobs_ mean (l)**	**s.d.**
12	565	48	12 671	233	138	92	0.57	0.25	92.3	10.5
24	923	112	24 535	3993	107	52	0.67	0.21	89.3	9.78
48	1327	236	51 205	11 040	111	54	0.67	0.19	99.3	46.2
96	3567	321	88 407	30 511	182	94	0.48	0.21	102.8	7.9
110	3600	1420	77 885	33 802	118	87	1.0	0.64	117.9	45.9
120	4823	1022	171 011	34 890	160	80	0.41	0.16	82.4	25.9
130	5768	3071	164 685	88 378	131	40	0.41	0.28	63.0	16.05
150	5760	1352	204 201	31 883	171	49	0.41	0.16	91.3	24.2

s.d.=standard deviation, AUC_last_=area under the dose concentration curve to last time point, AUC_inf_=area under the dose concentration curve extrapolated to infinity, *t*_1/2*γ*_=terminal half-life, Cl_obs_=observed clearance, V_ssobs_=volume of distribution at steady state. AUC_inf_=area under the dose concentration curve extrapolated to infinity is not given as this is an inappropriate and unreliable parameter when considering bound drug (total platinum).

for ultrafiltrate (free) and plasma (total) platinum, respectively. Samples were obtained from 41 of 42 patients. The ultrafiltrable platinum AUC increased linearly with dose (*r*^2^=0.79 *P*<0.0001) (Figure 2

**Figure 2:**
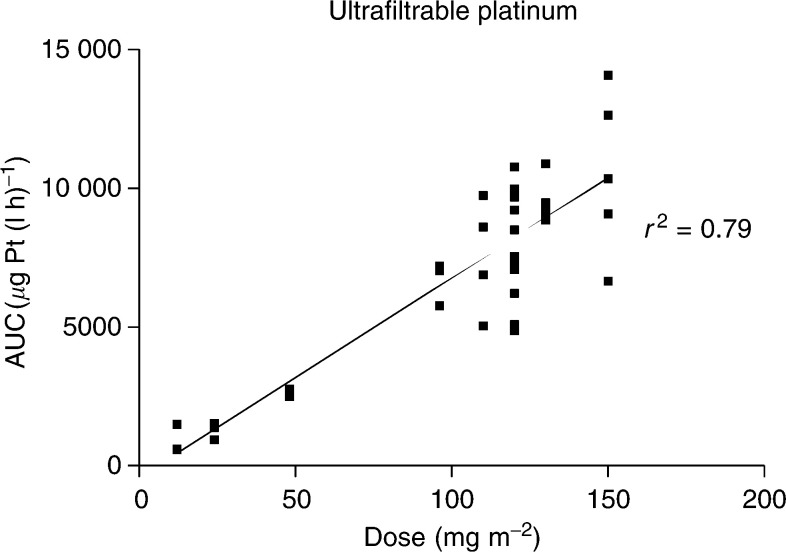
AUC ultrafiltrable platinum *vs* dose (mg m^−2^).

). There was one outlier, patient 24 treated at 120 mg m^−2^, with an AUC_last_=26 629 h *μ*g l^−1^ (data point not shown on graph as outside the range). Although at screening, this patient was eligible for the trial, when his serial antecedent results were reviewed it was clear that his renal function was fluctuating. His renal function was probably suboptimal following drug administration, resulting in markedly elevated AUC for free platinum compared with patients treated at the same dose level.

**Figure 3:**
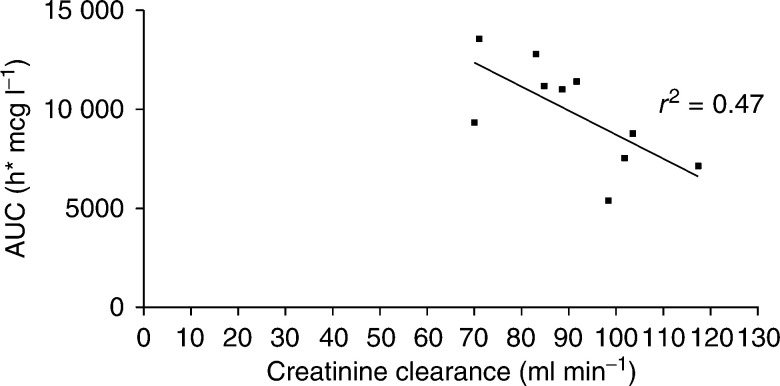
AUC ultrafiltrable platinum *vs* calculated creatinine clearance at 120 mg m^−2^.

The *C*_max_ (maximum concentration) also increased linearly with dose. The mean terminal half-life (*t*_1/2_) for free platinum was 85 h at dose level 120 mg m^−2^, which was longer than that seen in mice (10 h). At lower dose levels, the *t*_1/2_ was also apparently lower, attributable to the insensitivity of the pharmacokinetic assay and underestimation of plasma concentrations at later time points. The mean proportion of free platinum excreted in the urine was 32.9% (±15.9 s.d.) overall over 36 h (34.2±10.3% at 120 mg m^−2^). At 120 mg m^−2^, there was a moderate negative correlation between the ultrafiltrable platinum AUC and calculated creatinine clearance. (*r*^2^=0.47, *P*<0.0001) (Figure 3

).

**Figure 4:**
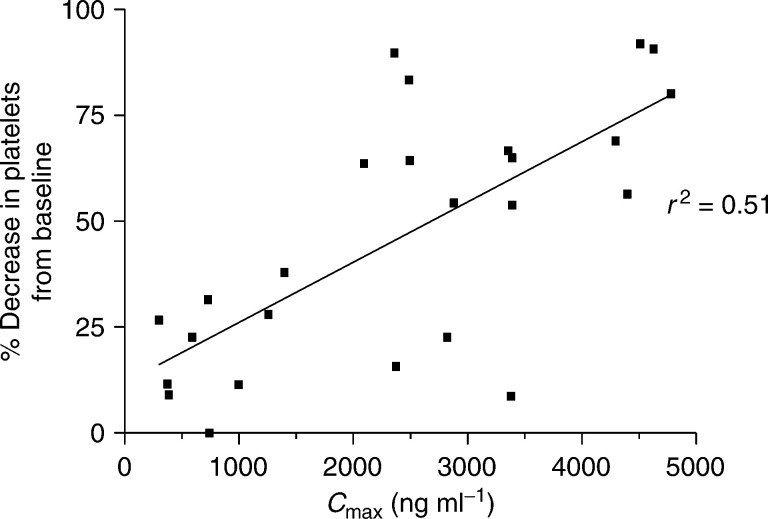
Decrease in platelets *vs C*_max_ free platinum.

There was no significant correlation between the percentage decrease of platelets or neutrophils with either AUC or *C*_max_ of free platinum. The closest relation appears to be between *C*_max_ for free platinum and the percent decrease in platelets (*r*^2^=0.51, *P*<0.0001) (Figure 4

).

### Antitumour activity

The best response to treatment in all patients is listed in Table 6

**Table 6:** Dose escalation – listing of dose levels treated, cycle length, and incidence of DLT

**Best response**	**No. of patients (%)**
Partial response	1 (2.4)
Stable disease	6 (14.3)
Early progression	3 (7.1)
Progressive disease	12 (28.6)
Not evaluable	19 (45.2)
Not assessed	1 (2.4)

. One patient with carcinoma of the ovary showed a radiological partial response after four cycles of treatment associated with a fall in her CA125 from 440 to 16 U ml^−1^. This patient was particularly heavily pretreated and, because of thrombocytopenia, her first cycle of the study drug was given at 150 mg m^−2^, a second cycle at 120 mg m^−2^, a third cycle at 96 mg m^−2^, followed by her fourth to eighth cycles, inclusive, at 80 mg m^−2^. Another patient with ovarian cancer had a partial response after two cycles, but this was not confirmed with a second CT scan, although there was a sustained >50% reduction in CA125. Five other patients had stable disease after multiple cycles of treatment; these patients had mesothelioma, ovarian carcinoma, nonsmall cell lung cancer, and melanoma. A further patient with mesothelioma, whose disease was not evaluable, experienced significant clinical benefit. This was demonstrated by a reduction in his pain and an improvement in respiratory and effort capacity. The combined effect of this allowed him to return to work.

## DISCUSSION

This study shows that AMD473 is devoid of significant organ-specific toxicity, and has predictable, dose-limiting myelosuppression and linear pharmacokinetics, as predicted by preclinical studies. The MTD is 150 mg m^−2^, with myelosuppression, particularly thrombocytopenia, being dose-limiting. The dose recommended for second-line treatment in a phase II study is 120 mg m^−2^ given every 21 days, based on expanded accrual in a less heavily pretreated population. However, our results show that two factors play an important role in determining the degree of toxicity observed, namely the extent of prior myelosuppressive chemotherapy and the performance status. As a consequence, there will be a group of patients (i.e. those who are relatively chemo-naive with ECOG performance status 0, 1) who will be able to tolerate higher doses (e.g. 150 mg m^−2^ q Q621 days) with minimal added toxicity. Conversely, those patients who have received extensive prior chemotherapy and/or have a performance status of ⩾2 may not be able to receive the drug on a 3-week schedule. This recommendation has been confirmed in the clinical experience seen in the Phase II setting to date. Escalation to 150 mg m^−2^ every 21 days has proved deliverable in patients with pretreated small cell, breast and ovarian cancers, but who are still relatively chemo-naive. However, the degree of myelosuppression at this dose level is higher and requires surveillance, as dose modifications are not infrequent (Bonhomi *et al*, 2001; Vandenberg *et al*, 2001; Gore *et al*, 2002).

There was a linear relation between dose and both *C*_max_ and AUC for ultrafiltrable and total platinum. Renal excretion was approximately 30%. Referring back to the preclinical models, the AUC (ultrafiltrate) for the mouse at the MTD (45 mg kg^−1^≈135 mg m^−2^) was 9844 *μ*g Pt(l h)^−1^ (Pre-clinical report CR UK, 1997). This is very close to that seen at 130 mg m^−2^ in the current study in man, with an AUC (ultrafiltrate) of 10 407 *μ*g Pt (l h)^−1^ (ratio 0.95). The ratio is similar to that of other platinum species and suggests that the method of dose escalation used in this study was appropriate for AMD473. It also gives confidence that the exposure achieved in man (and therefore potential anticancer activity) is equivalent to that seen in the mouse models (Raynaud *et al*, 1997). In contrast to some other recently investigated novel platinum compounds such as satraplatin, AMD473 is not subject to extensive degradative metabolism (McKeage *et al*, 1995; Raynaud *et al*, 1996; Judson *et al*, Q22000).

No clear relation could be shown between any pharmacokinetic parameters and toxicity other than a weak correlation between thrombocytopenia and peak platinum concentration achieved. This is in line with the findings reported by Stevenson *et al* (2001). Using an alternative, arguably more specific pharmacokinetic assay – stable isotope dilution liquid chromatography/tandem mass spectrometry, evidence for a pharmacokinetic/pharmacodynamic relation following AMD473 intravenous infusion was limited other than some evidence for a correlation between thrombocytopenia and AUC_(0−*t*)_ (Stevenson *et al*, 2001). Atomic absorption spectrophotometry was used in our current study as this was the method used in the preclinical work with AMD473. As is the case with many Phase I studies, the sensitivity of the assay is stretched at the lower dose levels dictated by a safe starting dose, but with an LLQ of 5 ng ml^−1^ the assay was considered to be more than sensitive enough to measure the study compound at therapeutic levels and to perform pharmacokinetically guided dose escalation.

The toxicity profile was similar to that of carboplatin, rather than cisplatin, and toxicity did not appear to be cumulative, with the possible exception of anaemia. The degree and duration of thrombocytopenia and neutropenia were predictable. Emesis appears to be moderate and can be controlled with a 5-HT3 antagonist and dexamethasone.

The DLT, not unexpectedly, proved to be myelosuppression with thrombocytopenia predominating. The patient who died from rectal bleeding while thrombocytopenic was felt to have died from disease progression rather than drug toxicity. However, a second patient did withdraw from treatment owing to similar toxicity. Neutropenia also occurred, commonly following the development of thrombocytopenia. Delay in neutrophil recovery did lead to dose delay in some patients. However, there was only one episode of neutropenic sepsis requiring hospitalization, occurring in a patient with a long-standing ureteric stent.

There was no evidence of ototoxicity (as assessed by audiogram) or symptomatic neurotoxicity, although some patients reported a change in taste, characteristically metallic in nature. Formal nerve conduction studies were not performed. No deterioration in serum creatinine or in calculated creatinine clearance was observed; however, serial ^51^Cr EDTA evaluations were not performed.

There was evidence of activity in ovarian cancer despite prior therapy with platinum. Preclinical studies suggested that AMD473 could overcome acquired resistance to platinum complexes. Certainly in this Phase I trial there was evidence of antitumour activity in a relatively heavily pretreated patient population. Early results from Phase II investigations conducted in nonsmall cell lung cancer, small cell lung cancer, and ovarian cancer suggest that AMD473 does have activity in patients who have received prior chemotherapy, including those who have received platinum-containing regimens (Bonomi *et al*, 2001; Quoix *et al*, 2001; Q3Gore *et al*, 2002). The clinical utility of this activity will require further clinical trials in appropriate patient populations. In such studies, it will be important to maximise treatment intensity relevant to the population under evaluation.
